# HIV Infection of Monocytes-Derived Dendritic Cells Inhibits Vγ9Vδ2 T Cells Functions

**DOI:** 10.1371/journal.pone.0111095

**Published:** 2014-10-23

**Authors:** Alessandra Sacchi, Alessandra Rinaldi, Nicola Tumino, Rita Casetti, Chiara Agrati, Federica Turchi, Veronica Bordoni, Eleonora Cimini, Federico Martini

**Affiliations:** 1 Laboratory of Cellular Immunology, National Institute for Infectious Diseases “Lazzaro Spallanzani”, Rome, Italy; 2 Department of Biology, University of Rome “Tor Vergata”, Rome, Italy; Temple University School of Medicine, United States of America

## Abstract

DCs act as sentinel cells against incoming pathogens and represent the most potent antigen presenting cells, having the unique capability to prime naïve T cells. In addition to their role in induction of adaptive immune responses, DC are also able to activate innate cells as γδ T cells; in particular, a reciprocal crosstalk between DC and γδ T cells was demonstrated. However, whether HIV infection may alter DC-Vγ9Vδ2 T cells cross-talk was not yet described. To clarify this issue, we cultured activated Vγ9Vδ2 T cells with HIV infected monocyte derived DC (MoDC). After 5 days we evaluated MoDC phenotype, and Vγ9Vδ2 T cells activation and proliferation. In our model, Vγ9Vδ2 T cells were not able to proliferate in response to HIV-infected MoDC, although an up-regulation of CD69 was observed. Upon phosphoantigens stimulation, Vγ9Vδ2 T cells proliferation and cytokine production were inhibited when cultured with HIV-infected MoDC in a cell-contact dependent way. Moreover, HIV-infected MoDC are not able to up-regulate CD86 molecules when cultured with activated Vγ9Vδ2 T cells, compared with uninfected MoDC. Further, activated Vγ9Vδ2 T cells are not able to induce HLA DR up-regulation and CCR5 down-regulation on HIV-infected MoDC. These data indicate that HIV-infected DC alter the capacity of Vγ9Vδ2 T cells to respond to their antigens, pointing out a new mechanisms of induction of Vγ9Vδ2 T cells anergy carried out by HIV, that could contribute to immune evasion.

## Introduction

Dendritic cells (DC), characterized as the most potent antigen-presenting cells (APC), represent a multi-functional population of cells. In steady-state conditions, DC are in an immature stage and induce tolerogenic T cell responses [1], [2]. In an inflammatory microenvironment, upon ligand recognition by Toll Like Receptors (TLR), maturation process occurs and DC migrate to the lymph nodes where productive adaptive immune responses are induced [3], [4]. In addition to their role in induction of adaptive immune responses, DC are also able to activate innate cells as natural killer (NK) cells [5] and γδ T cells; in particular, a reciprocal crosstalk between DC and γδ T cells was demonstrated [6], [7]. In human peripheral blood, the predominant subset expresses the Vδ2 chain associated with Vγ9 (Vγ9Vδ2 T cells) and represents 70% of circulating γδ T cells in adults. Vγ9Vδ2 T cells respond to non-processed and non-peptidic phosphoantigens in an HLA-unrestricted manner [8], in particular, it has been recently demonstrated that Vγ9Vδ2 T cells are activated by phosphoantigen presented by butyrophilin 3A [9]. Circulating Vγ9Vδ2 T cells represent a large and broadly reactive population that rapidly responds to the presence of microbial invaders [10]. Invading pathogens have the specific ability to directly elicit a strong Vγ9Vδ2 T cell response in the early phases of infection, leading to the synthesis of soluble factors (cytokines and chemokines), that orchestrate the specific adaptive immune response, and directly interfering with the infection spread by exerting a potent cytotoxic activity. It has been shown a bidirectional activating interaction between DCs and activated Vγ9Vδ2 T cells [7]. However, some pathogen, as Mycobacterium tuberculosis, may alter the activation of Vγ9Vδ2 T cells [11], contributing to bacterial immune escape.

HIV infection deeply affects several issues of immune response including DCs [12] and Vγ9Vδ2 T cells [13], contributing to the loss of immune competence.

Studies of HIV-1 infected humans suggest that HIV infection can impact on repertoire and effector function of Vγ9Vδ2 T cells. The frequency of Vγ9Vδ2 T cells is markedly reduced in the blood of HIV-1-infected humans [4]–[16]. Moreover, the remaining Vγ9Vδ2 T cells are unable to perform their effector function, with a reduced production of IFN-γ and TNF-α, and unable to expand after TCR stimulation [16]. The cytolytic function of Vγ9Vδ2 T cells is also impaired during HIV-1 infection [17]. The molecular mechanisms causing anergy to TCR triggering are still under scrutinity; we previously reported a decreased expression of CD3ζ TCR-associated molecule on Vγ9Vδ2 T cells from HIV infected patients, that correlates with their reduced functionality [18]. It has been also showed a specific depletion of Vγ2-Jγ1.2 T cells, that could contribute to the loss of phosphoantigen response capability [19]. The ability of DC to potentiate Vγ9Vδ2 T cells production of inflammatory cytokines required for their own maturation was clearly demonstrated, but whether HIV infection may impair DC-Vγ9Vδ2 T cells cross-talk was not yet described. Understanding this issue could be useful for the comprehension of the strategies used by HIV to evade immune system, and in designing therapeutic approaches targeting both populations. Aim of the present work was to evaluate whether HIV infection may alter the cross-talk between DC and Vγ9Vδ2 T cells. We show that HIV infection of monocytes-derived DC (MoDC) drastically affects the capacity of Vγ9Vδ2 T cells to respond to Isopentenyl pyrophosphate (IPP), and to induce MoDC maturation, thus revealing a new mechanism that could contribute to Vγ9Vδ2 T cells anergy observed in HIV+ patients.

## Materials and Methods

### DC preparation and infection

Anonymous buffy coats of healthy donors were obtained from the Transfusion Center of San Camillo hospital (Dipartimento Interaziendale Territoriale di Medicina Trasfusionale LAZIO OVEST, www.sancamilloforlanini.rm.it). Peripheral blood mononuclear cells (PBMCs) were isolated by density gradient centrifugation using Lympholyte-H (CEDERLANE, Canada). Monocytes were positively separated by anti-CD14 magnetic beads (MACS, Milteny Biotec, Germany), according to manufacturer’s instructions. CD14+ cells (mean purity 95%) were then resuspended in RPMI 1640 (EuroClone, UK) supplemented with 10% heat-inactivated defined fetal bovine serum (FBS) (HyClone), 2 mM L-Glutammin, 10 mM HEPES buffer (N-2-hydroxyethylpiperazine-N-2-ethanesulfonic acid), 2 mM penicillin, and 50 µg/mL streptomycin (EuroClone, UK). MoDCs were differentiated from monocytes as previously described [20]; briefly, monocytes (1×10^6^/ml) were cultured for 5 days in the above described medium in the presence of 50 ng/mL granulocyte-macrophage colony-stimulating factor (GM-CSF) and 10 ng/mL interleukin-4 (IL-4) (Prospec, Israel). After 5 days MoDC were infected with HIV_BAL_ (200 ng of p24/3×10^6^ cells) for 3 hours, then extensively washed and cultured in the above described medium (1×10^6^/ml) (without cytokines) for 18 hours.

### γδ T cells purification and MoDC co-culture

γδ T cells were positively separated from PBMC by anti-TCR γδ microbead kit (MACS, Milteny Biotec, Germany), according to manufacturer’s instructions. Purified γδ T cells (mean purity 90%) were frozen until DC differentiation. Purified γδ T cells were thawed 18 hours before activation and cultured in the above described medium (2×10^6^/ml). After recovering, γδ T cell viability was determined by trypan blue exclusion (>80%) and then they were co-cultured with HIV-infected MoDC (0.5×10^6^ MoDC+0.5×10^6^ γδ T cells in 1 ml of above described medium, without cytokines) and activated with isopentenylpyrophosphate (IPP) for 5 days. In selected experiments, γδ T lymphocytes were physically separated from DC by a semi-permeable membrane (transwell, 6.5-mm of diameter, 0.4- µm pore size in 24-well plates; Costar). The lower compartment of the wells contained.

MoDC (0.5×10^6^ cells); the upper compartments (transwell insert) contained γδ T lymphocytes (0.5×10^6^ cells). After 5 days, culture supernatants were collected and stored at-80°C, and cell phenotype were evaluated by flow cytometry. Where indicated γδ T cells were CFDA-SE labeled (10 µM, Invitrogen) according to manufacturer’s instructions. After 5 days, the proliferation rate was evaluated by flow cytometry.

### Flow cytometry

The following monoclonal antibodies were used to characterize MoDC: anti-CD86 FITC, anti-CD1a PE, anti-HLA-DR PERCP, anti-CD83 APC, anti-CD14 APC-H7, anti-CD80 FITC, anti-CD40 PE, anti-HLA-I APC, anti-CCR7 PE-Cy7, anti-CCR5 APC-H7 from BD Biosciences, and anti-BT3A.1 PE from BioLegend. To evaluate γδ T lymphocytes phenotype we used anti-Vδ2 FITC, anti-CD3 PE, anti-CD69 PERCP, anti-CD45RA PE-Cy7, anti-CD27 APC, anti-CD16 PACIFIC BLU, anti-CD25 APC-H7 (BD Biosciences). In brief, the cells were washed twice in PBS, 1% BSA, and 0.1% sodium azide, and were stained with the mAbs for 15 min at 4°C. The cells were then washed and fixed with 4% paraformaldehyde, and analyzed using a FACS Canto II (Becton Dickinson). Since the presence of 2 purified populations, the gating strategy was performed as follow: dead cells were excluded by scatter characteristics; MoDC were identified by morphological parameters (FSC vs SSC); gated cells were then analyzed for the expression of the molecules described above. T lymphocytes were first gated by using morphological parameters; in this gate Vγ9Vδ2 T cells were identified as Vδ2+CD3+. Analysis was carried out by using Facs Diva software (Becton Dickinson). The histogram overlays were performed by FlowJo software (TreStar, Olten, Switxìzerland).

### Analysis of cytokines production and HIV replication

Cytokines released in the supernatants were analyzed by using a customized Bio-Plex Pro Assay (BIO RAD) according to manufacturer’s instructions. The assay was able to evaluate the following cytokines and chemokines: IL-1β, IL-2, IL-4, IL-5, IL-6, IL-7, IL-8, IL-10, IL-12(p70), IL-13, G-CSF, IFN-γ, MCP-1, MIP-1b, RANTES, TNF-α, GM-CSF, IL-17.

HIV replication was evaluated by measuring p24 released in supernatants by a microelisa system (Vironostika HIV-1 antigen, Biomarieux) according to manufacturer’s instructions.

### Statistical analysis

Results were evaluated using Mann-Whitney test. A p value <0.05 was considered statistically significant. GraphPad Prism software (version 4.00 for Windows; GraphPad) was used to perform the analysis.

## Results

### HIV-infected MoDC inhibit Vγ9Vδ2 T cell proliferation

**Figure 1:**
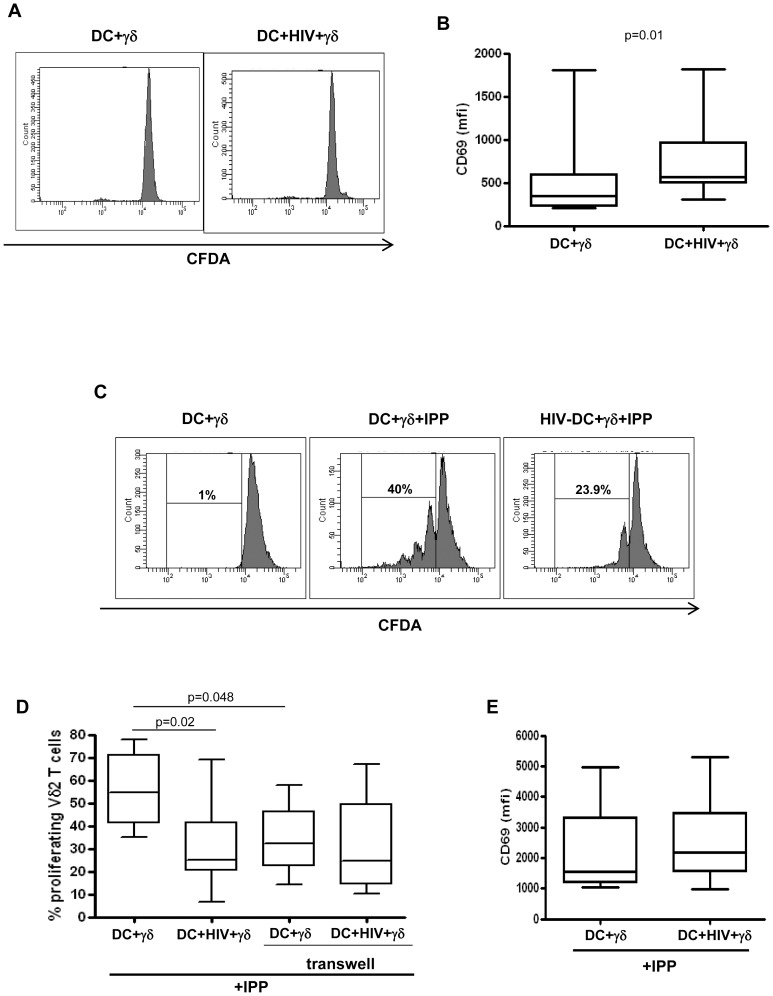
Effects of HIV-infected MoDC on Vγ9Vδ2 T cells proliferation. MoDC were infected with HIV_BAL_ and cultured with CFDA-SE labeled Vγ9Vδ2 T cells. After 5 days, Vγ9Vδ2 T cells proliferation and activation was evaluated by flow cytometry. (A) Representative histogram plots of one out of seven independent experiments showing Vγ9Vδ2 T cells proliferation. (B) CD69 expression on Vγ9Vδ2 T cells in the indicated conditions. Vγ9Vδ2 T cells labeled with CFDA-SE were stimulated with IPP in the presence of MoDC infected or not with HIV_BAL._ After 5 days, Vγ9Vδ2 T cells proliferation was evaluated by flow cytometry. (C) Representative histogram plots of one out of seven independent experiments showing Vγ9Vδ2 T cells proliferation. (D) Percentage of proliferating Vγ9Vδ2 T cells upon IPP stimulation in the indicated conditions. (E) CD69 expression (mean fluorescence intensity, mfi) on IPP stimulated Vγ9Vδ2 T cells cultured with HIV infected or uninfected MoDC. Results are shown as Box and Whiskers: the box encompasses the interquartile range of individual measurements, the horizontal bar-dividing line indicates the median value, and the whiskers represents maximum and minimum values.

**Figure 2:**
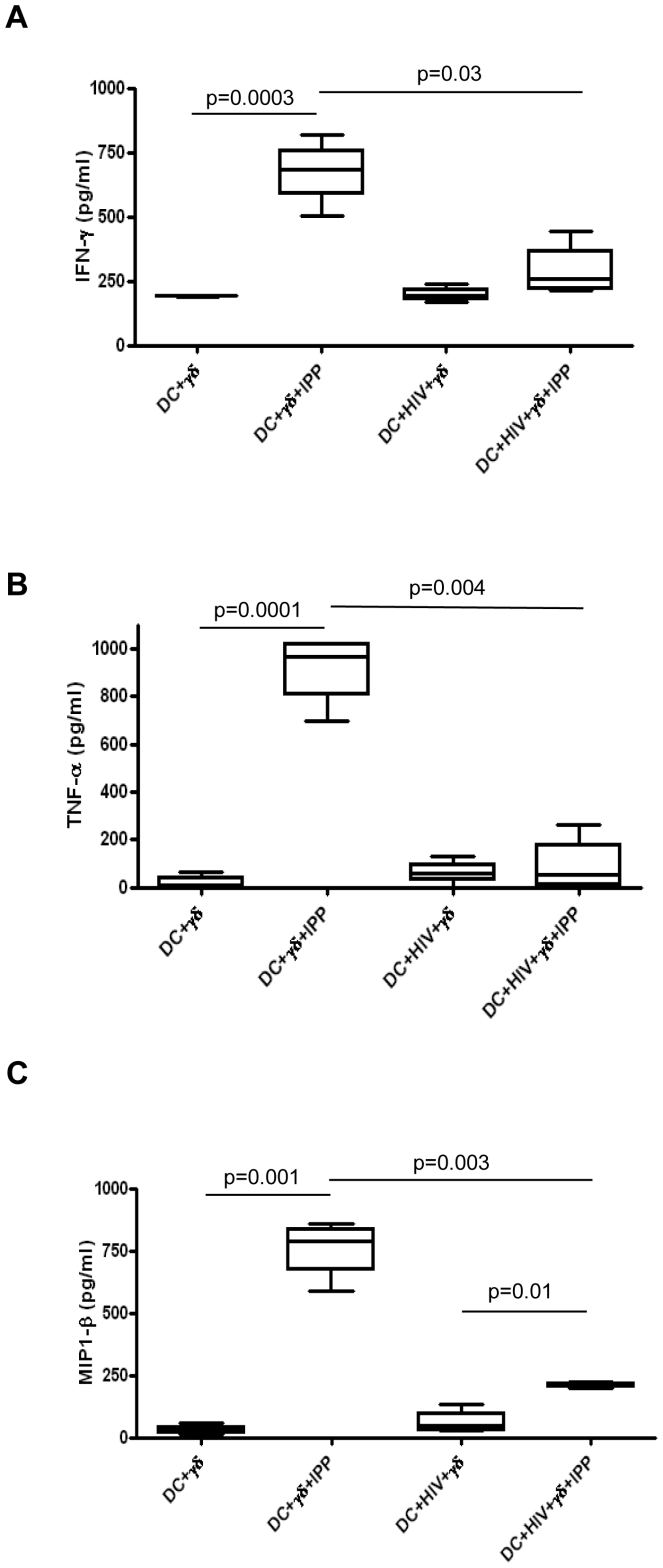
HIV-infected MoDC inhibit Vγ9Vδ2 T cells cytokines production. Vγ9Vδ2 T cells were stimulated with IPP in the presence of MoDC infected with HIV_BAL._ After 5 days, cytokines released in the supernatants were evaluated by a multiplex immunoassay. (A) IFN-γ, (B) TNF-α, and (C) MIP1-β production, in seven independent experiments, are shown as Box and Whiskers: the box encompasses the interquartile range of individual measurements, the horizontal bar-dividing line indicates the median value, and the whiskers represents maximum and minimum values.

Vγ9Vδ2 T cells are able to recognize small non peptidic, phosphorilated antigens derived from some bacteria or altered metabolic cell cycles [8]. We asked whether HIV-infected MoDC are capable to activate Vγ9Vδ2 T cells, in terms of CD69 expression and proliferation. To answer this issue, MoDC were infected with HIV (200 ng p24/3×10^6^ MoDC), and cultured with purified γδ T cells, stained with CFDA-SE, for 5 days, and Vγ9Vδ2 proliferation was assessed by flow cytometry. Figure 1A shows that HIV-infected MoDC did not induce Vγ9Vδ2 T cell proliferation. However, we found an up-regulation of CD69 on the membrane of Vγ9Vδ2 T cultured with HIV-infected MoDC compared to uninfected cells (fig. 1B), indicating that some activation occurred. On the other hand, we evaluated the effect of HIV-infected MoDC on the Vγ9Vδ2 T cell proliferation rate induced by IPP. To this aim, MoDC were infected with HIV (200 ng p24/3×10^6^ MoDC), and cultured with purified γδ T cells, stained with CFDA-SE, and activated with IPP at the time of co-culture. After 5 days, Vγ9Vδ2 proliferation was assessed by flow cytometry. HIV-infected MoDC strongly inhibit IPP-induced Vγ9Vδ2 T cells proliferation (fig. 1C and D). No differences in CD69 expression was pointed out (fig. 1E). Then, we evaluated whether the inhibition of Vγ9Vδ2 T cells proliferation by HIV-infected MoDC is cell contact dependent. To this aim we cultured HIV-infected MoDC and Vγ9Vδ2 T cells physically separated by a semi-permeable membrane, and after 5 days Vγ9Vδ2 T proliferation was tested by flow cytometry. As previously demonstrated [21], [22], we confirmed that Vγ9Vδ2 T cell proliferation is significantly decreased when cell contact with MoDC was prevented; however, when MoDC and Vγ9Vδ2 T cells were physically separated by transwell, HIV infection did not inhibit Vγ9Vδ2 T cell proliferation (fig. 2B), suggesting that MoDC- Vγ9Vδ2 T cell contact is central to induce HIV-related Vγ9Vδ2 T cells impairment.

We also analyzed the differentiation profile of Vγ9Vδ2 T cells by the expression of CD45RA and CD27 (Naïve: CD45RA+CD27+, Central Memory (CM): CD45RA-CD27+, Effector Memory (EM): CD45RA-CD27-, Effector (EFF): CD45RA+CD27-) after culture with HIV-infected MoDC. We found that, as expected, in all conditions 60–80% of Vγ9Vδ2 T cells were CM, 10–40% EM, and the remaining fraction were naïve and EFF. Moreover, HIV-infected MoDC did not induce any alteration of the Vγ9Vδ2 T cells differentiation phenotype, in both IPP stimulated and not stimulated conditions (data not shown).

### HIV-infected MoDC inhibit cytokine production by activated Vγ9Vδ2 T cells

Since we observed an inhibition of Vγ9Vδ2 T cells proliferation after antigen stimulation when cells are cultured with HIV-infected DC, we wondered if cytokines production was also impaired. To this end, HIV-infected MoDC were cultured with Vγ9Vδ2 T cells activated with IPP at the beginning of the co-culture, and after 5 days of culture supernatants were collected and tested for 18 cytokines. When Vγ9Vδ2 T cells were cultured with HIV-infected DC, an inhibition of IFN-γ, TNF-α and MIP1-β production was observed compared to the culture with uninfected DC (Fig. 2), while other cytokines were not detected (IL-1β, IL-2, IL-4, IL-5, IL-7, IL-10, IL-12(p70), IL-13, G-CSF, MCP-1, GM-CSF, IL-17) or no differences were observed (IL-6, IL-8, RANTES) (data not shown). These data indicate that HIV-infected MoDC impaired the capacity ofVγ9Vδ2 T cells to produce pivotal cytokines upon antigen stimulation.

### Human Vγ9Vδ2 T cells fails to inhibit HIV replication in MoDC

**Figure 3:**
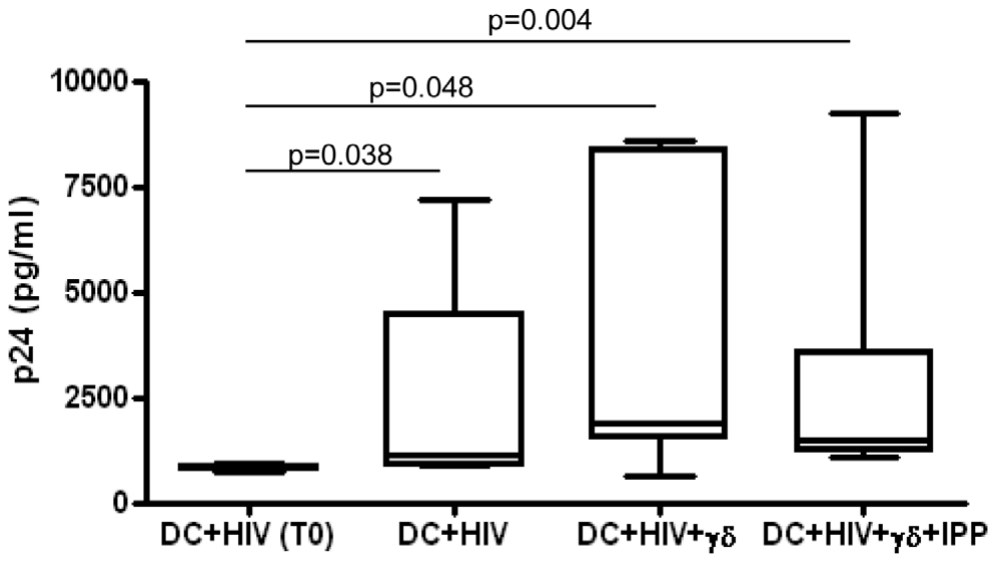
Vγ9Vδ2 T cells do not inhibit HIV replication in MoDC. MoDC were infected with HIV_BAL_ and cultured with purified γδT cells stimulated or not stimulated with IPP. Before culture with γδT cells (DC+HIV T0), and after 5 days HIV p24 protein was tested in the supernatants by ELISA. Results from seven independent experiments are shown as Box and Whiskers: the box encompasses the interquartile range of individual measurements, the horizontal bar-dividing line indicates the median value, and the whiskers represents maximum and minimum values.

It was previously shown that activated Vγ9Vδ2 T cells inhibit HIV replication in target cells, mostly by producing chemokines such as RANTES and MIP1-β [23] and by lysing infected cells. However, there are no data on their effects on HIV replication in MoDC. Since we observed that HIV infected MoDC inhibit Vγ9Vδ2 T cells proliferation and cytokines production induced by IPP, we asked whether Vγ9Vδ2 T cells maintain their capability to inhibit HIV replication in MoDC. To answer this issue, we tested the amount of p24 in the supernatants of HIV infected MoDC cultured for five days with Vγ9Vδ2 T cells activated or not with IPP. Figure 3 shows that the level of p24 released by HIV-infected MoDC increased after 5 days of culture compared to p24 amount detected soon after infection (T0). Moreover, p24 released by HIV-infected MoDC cultured with activated or not activated Vγ9Vδ2 T cells is comparable to those produced by HIV-infected MoDC alone (p = 0.141, p = 1.000 respectively),suggesting a complete ineffectiveness of Vγ9Vδ2 T cells towards HIV infected MoDC.

### Butyrophilin 3A1 is not involved in Vγ9Vδ2 T cells inhibition

**Figure 4:**
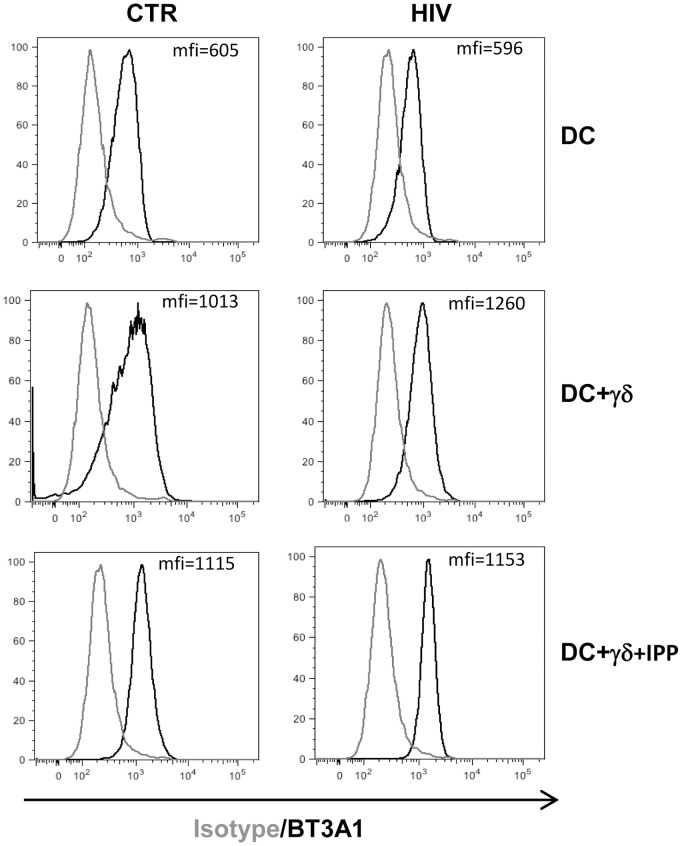
Butyrophilin 3A1 is not involved in Vγ9Vδ2 T cells inhibition. MoDC were infected with HIV_BAL_ and cultured with purified γδT cells stimulated stimulated with IPP. After 5 days the expression of BT3A1 was analyzed by flow cytometry. One representative of 3 independent experiments is shown.

Our results indicate that HIV-infected MoDC inhibit the capacity of Vγ9Vδ2 T cells to respond to IPP. Recently, it has been clearly demonstrated that butyrophilin 3A1 (BT3A1) binds phosphorylated antigens and activates Vγ9Vδ2 T cells [9]. Thus, we wondered whether the observed Vγ9Vδ2 T cells functional impairment induced by HIV-infected MoDC was caused by a diminution of BT3A1 expression. To this aim, we analyzed the expression of BT3A1 on HIV-infected MoDC after 5 day of culture with or without Vγ9Vδ2 T cells by flow cytometry. We found that after 5 days of infection BT3A1 expression on MoDC was comparable to uninfected cells. When cultured with Vγ9Vδ2 T cells, neither Vγ9Vδ2 T cells activation with IPP nor HIV infection induced a modulation of BT3A.1 (fig. 4). This data suggest that the inability of Vγ9Vδ2 T cells to be activated by IPP when co-cultured with HIV-infected MoDC seems not due to a loss of BT3A.1 expression.

### Vγ9Vδ2 T cells failed to induce the maturation of HIV-infected MoDC

**Figure 5:**
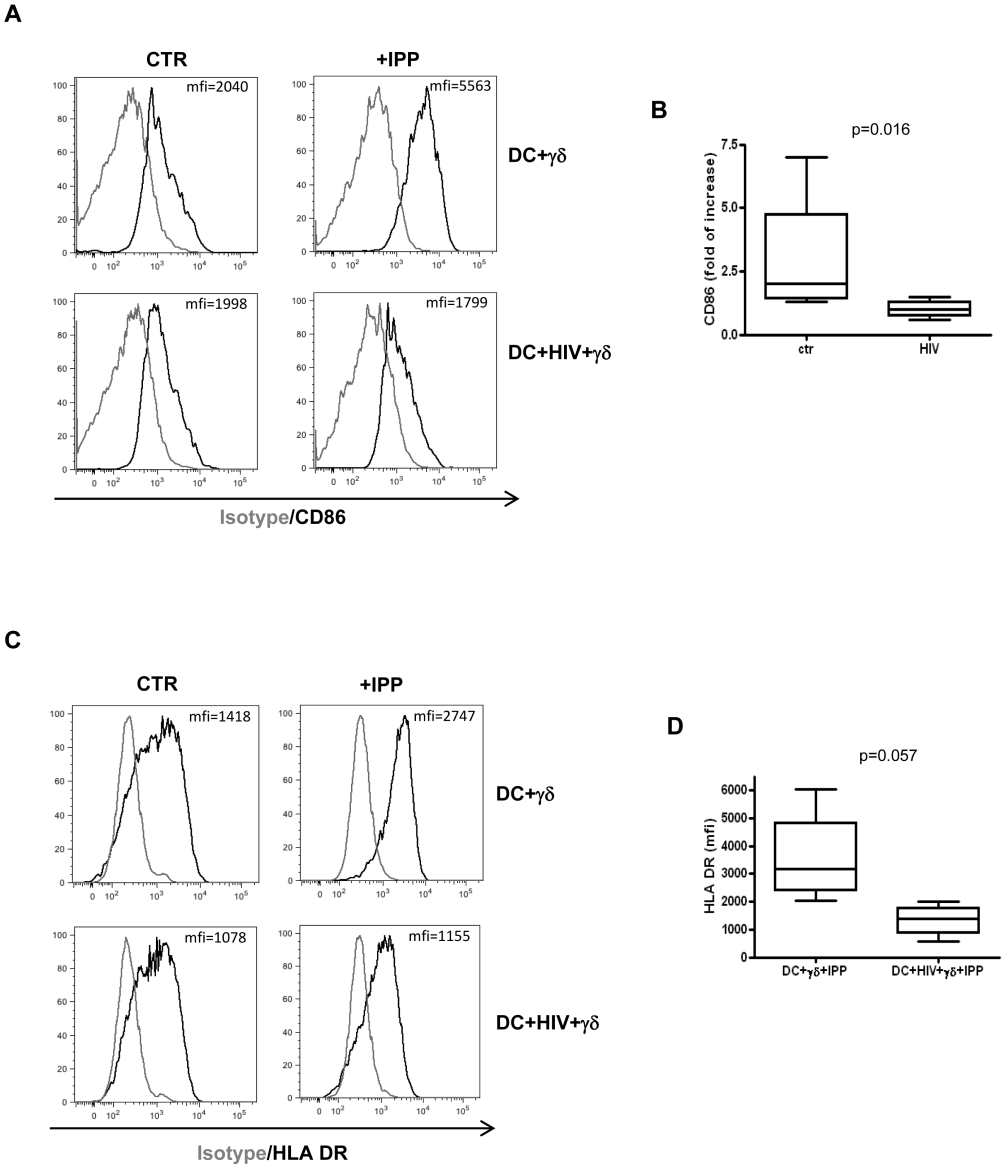
Vγ9Vδ2 T cells fail to induce CD86 and HLA-DR up-regulation on HIV-infected MoDC. MoDC were infected with HIV_BAL_ and cultured with purified γδT cells for 5 days. Then, MoDC phenotype were evaluated by flow cytometry. (A) Representative histogram plots of one out of seven independent experiments showing CD86 expression on MoDC in the indicated conditions. (B) Induction of CD86 expression on MoDC by activated Vγ9Vδ2 T cells (fold of increase: IPP stimulated/not stimulated). (C) Representative histogram plots of one out of four independent experiments showing HLA-DR expression on MoDC in the indicated conditions. (D) HLA-DR expression on MoDC (mfi) in the indicated conditions. Results are shown as Box and Whiskers: the box encompasses the interquartile range of individual measurements, the horizontal bar-dividing line indicates the median value, and the whiskers represents maximum and minimum values.

Previous studies demonstrated that activated Vγ9Vδ2 T cells are able to maturate DC [7], [21]; in particular, they induce the expression of the co-stimulatory molecule CD86 (B7.1) that plays a central role in Vγ9Vδ2 T cells proliferation. We asked whether the inhibition of Vγ9Vδ2 T cell proliferation was due to CD86 impairment on HIV-infected DC. To this aim MoDC were infected with HIV_BAL_, as described above, and cultured with purified, IPP activated γδ T cells (ratio 1∶1). Five days after, we evaluated MoDC phenotype by flow cytometry. In line with the Vγ9Vδ2 T cell cytokines production involved in the DC maturation, we found that, as previously described, activated Vγ9Vδ2 T cells were able to up-regulate of CD86 on MoDC; on the contrary, when cells were HIV-infected Vγ9Vδ2 T cells failed to induce the up-regulation of CD86 (fig. 5A and B).

**Figure 6:**
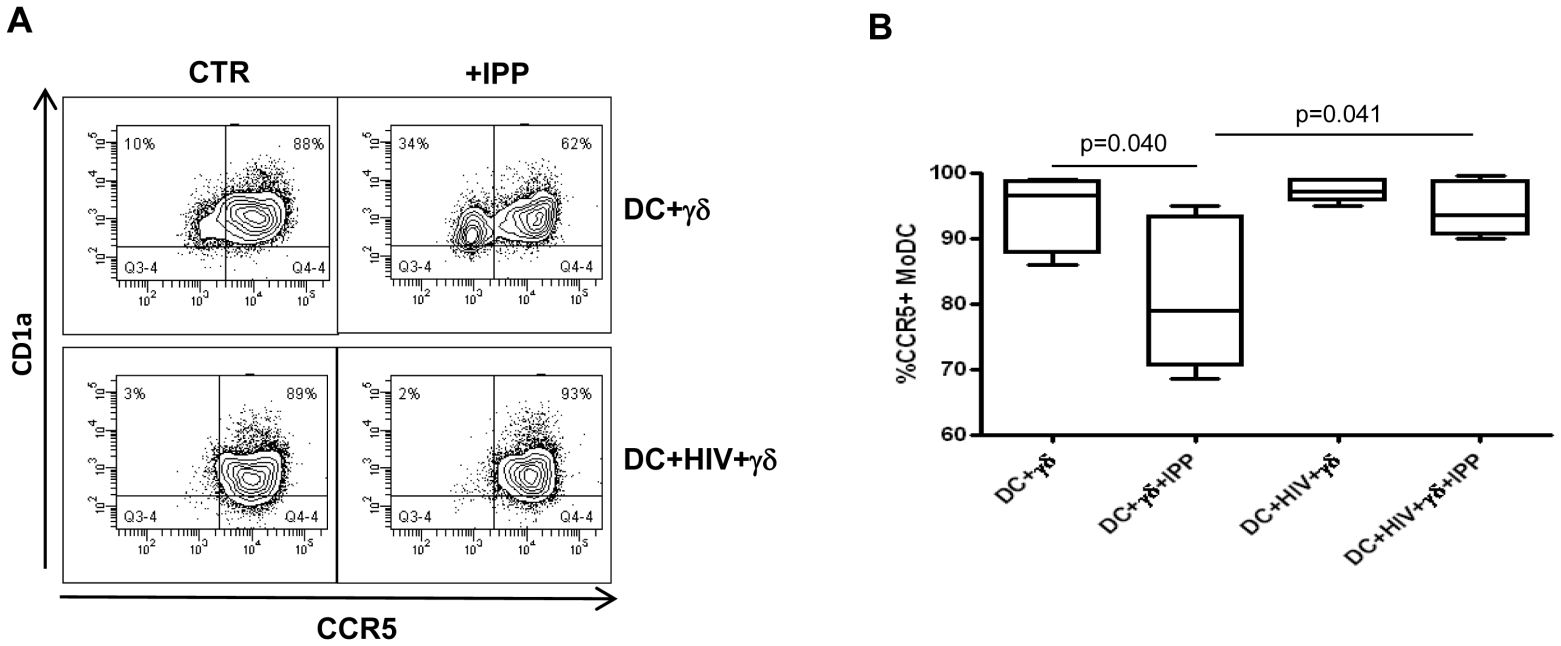
HIV-infected MoDC fail to down-regulate CCR5. MoDC were infected with HIV_BAL_ and cultured with purified γδT cells for 5 days. MoDC phenotype were evaluated by flow cytometry. (A) Representative histogram plots of one out of seven independent experiments showing CCR5 expression on MoDC in the indicated conditions. (B) Percentage of CCR5+ MoDC cultured with Vγ9Vδ2 T cells in the indicated conditions. Results are shown as Box and Whiskers: the box encompasses the interquartile range of individual measurements, the horizontal bar-dividing line indicates the median value, and the whiskers represents maximum and minimum values.

We also found that activated Vγ9Vδ2 T were not able to induce HLA-DR up-regulation on HIV-infected MoDC compared to uninfected cells (fig. 5C, D), albeit the difference is not statistically significant (p = 0.057). These data suggest that the inhibition of Vγ9Vδ2 T cells functions may alter the antigen presentation capacity of DC to CD4+ T cells. Finally, activated Vγ9Vδ2 T cells fail to down-modulate CCR5 expression on HIV-infected MoDC compared to uninfected cells (fig. 6), thus maintaining DC highly susceptible to HIV infection. We did not find any alterations for the other tested markers.

## Discussion

The concept of a strict dependent relationship between cells from innate and adaptive immunity changed the point of view about the regulation of immune system. During the most part of host reactions, both adaptive and innate sections cooperate in the host’s protection and tissue damage.

The innate cells recruited or resident in the tissues, and their interactions, play a crucial role in the containment of infection and the deployment of adaptive immune response [5]. In particular, DC, in addition to their role in induction of adaptive immune responses, are able to activate other innate immune cells [5]. The influence exerted by γδ T cells on DCs system was also demonstrated, showing that human Vγ9Vδ2 T cells activated in vitro by phosphoantigens are capable of inducing maturation of MoDCs, thus potentially enhancing their antigen presentation capability [24], [25]. In this paper we found that HIV infection alter DC- Vγ9Vδ2 T cell interactions by strongly inhibiting Vγ9Vδ2 T cell functions. As previously demonstrated [26], HIV infection of DC does not lead to MoDC maturation; we show that when they are infected with HIV, they cannot up-regulate CD86 upon stimulation with activated Vγ9Vδ2 T cells compared with uninfected MoDC. Moreover, HIV infection inhibits the Vγ9Vδ2T cells mediated down-modulation of CCR5 and up-regulation of HLA-DR on MoDC. Altogether these data suggest that HIV infection could increase the DC susceptibility to HIV infection, and interfere with DC antigen presentation to CD4 T cells, thus contributing to the impaired HIV-specific adaptive immune response. However, these issue needs further investigations. We also found that the incapacity of MoDC to be matured by Vγ9Vδ2 T cells was not due to a paralysis of the maturation machinery of MoDC, but rather to an altered capability of Vγ9Vδ2 T cells to respond to TCR triggering. In fact, Vγ9Vδ2 T cells proliferation upon IPP stimulation was inhibited when cultured with HIV infected MoDC by a mechanism that needs cell contact, suggesting that after HIV infection something occurs in DC that induced Vγ9Vδ2 T cells inability to respond to phosphoantigens. IFN-γ and TNF-α production was inhibited as well, thus decreasing potent signals by which Vγ9Vδ2 T cells ensure that DC maturation is skewed towards a Th1 response.

It was clearly demonstrated that DC- Vγ9Vδ2 T cells interaction needs cellular contact. In fact, the proliferation of γδ T cells induced by IPP, in the absence of IL-2, required the presence of a second signal mediated by DC through CD86. Further, DC potentiate Vγ9Vδ2 T cells activation and cytokines production [7], [21], [22]. Altogether these findings indicate that HIV-infected MoDC, inhibiting the production of IFN-γ and TNF-α by IPP stimulated Vγ9Vδ2 T cells, do not up regulate CD86, thus limiting Vγ9Vδ2 T cells proliferation. Moreover, we observed that activated Vγ9Vδ2 T cells were not able to inhibit HIV replication in MoDC, as demonstrated by the level of p24 released in the supernatants. Our data suggest that the incapacity of Vγ9Vδ2 T cells to maturate DC could render them more permissive to HIV replication [27]; moreover, the inhibition of MIP1-β production could contribute to augment HIV infection [28]. An efficient capacity of Vγ9Vδ2 T cells to inhibit HIV replication in PBMC from healthy donors was previously shown [23]; however, dendritic cells represent the 0.5–1% of PBMC, thus indicating that the type of infected cells interacting with Vγ9Vδ2 T cells could be important in determining the effects on this cell subset.

It was reported that during HIV infection the effector memory Vγ9Vδ2 T cells decreased [29], [30]; however, we did not found any alteration of Vγ9Vδ2 T cells differentiation phenotype, suggesting that more complex phenomena occurred *in vivo,* probably due to the general immune activation observed in HIV+ patients [31].

Data presented in this paper show that Vγ9Vδ2 T cells functions were severely inhibited by HIV-infected MoDC, however, the mechanism remains elusive. In fact, we did not observed a CD3ζ down-modulation in Vγ9Vδ2 T cells after culture with HIV infected MoDC (data not shown), as previously shown on Vγ9Vδ2 T cells from HIV infected patients [18]. Moreover, we found that HIV infection did not alter the expression of BTN3A on MoDC membrane, suggesting that the inhibition of Vγ9Vδ2 T cells response to IPP is not caused by a modulation of this molecule. Published reports suggested that phosphorylated antigens modify BTN3A molecules via interactions with the cytoplasmic regions, which would potentially involve dimerization and clustering [32], [33]. Thus, we cannot exclude that HIV infection may impair molecular modifications of BTNA3-IPP complexes that are not recognized by Vγ9Vδ2 T cells. More studies are needed to clarify the fine molecular events leading to Vγ9Vδ2 T cells activation and the possible role of HIV infection in the inhibition of such events.

To date, it is not clear whether there exist some HIV antigens or HIV-infected cells derived antigens able to activate Vγ9Vδ2 TCR. In our model, we could not see any proliferation of Vγ9Vδ2 T cells after culture with HIV infected MoDC, nor cytokines production; however, an augmented expression of CD69 was observed. CD69 is a type II transmembrane protein and a member of the C-type lectin-like receptor family that is expressed in leukocytes upon stimulation [34]. CD69 acts as a signal transducer in inflammatory processes. Several studies using models of inflammatory diseases point to an immunoregulatory role for CD69 during the immune response, suggesting that, probably depending on micro-environmental conditions, CD69 could exert different functions [35], [36], [37], [38]. Since our results indicate that HIV infection of MoDC impair Vγ9Vδ2 T cells activation, the observed CD69 up-regulation could suggest suppression rather than activation; however, this issue needs further investigations.

The inhibition of T cell functions by HIV-infected DC is not limited to γδ T cells. In fact, it has been shown that HIV infection of DC alter their capacity to stimulate αβ T cells [39], even if, this issue remains debated (reviewed in [40]). The infection of DC impair also the interplay with innate immune populations as NK cells [41], [42], indicating that among the strategies exploited by HIV to evade the immune response, the infection of DC deeply affects the functionality of different arms of immune system, thus contributing to HIV persistence.

In conclusion, we show herein for the first time that MoDC infected by HIV can alter the functional capabilities of Vγ9Vδ2 T cells through a cell contact dependent mechanism. These findings provide further evidences of the complex relationship between important players of innate immunity and its modulation by HIV, that has to be take into account in evaluating new therapeutic or vaccine strategies.
